# High fat diet-induced glucose intolerance impairs myocardial function, but not myocardial perfusion during hyperaemia: a pilot study

**DOI:** 10.1186/1475-2840-11-74

**Published:** 2012-06-20

**Authors:** Charissa E van den Brom, Carolien S E Bulte, B Margreet Kloeze, Stephan A Loer, Christa Boer, R Arthur Bouwman

**Affiliations:** 1 Department of Anesthesiology, VU University Medical Center, Boelelaan 1117, 1081 HV, Amsterdam, The Netherlands; 2 Laboratory for Physiology, VU University Medical Center, van der Boechorststraat 7, 1081 BT, Amsterdam, The Netherlands

**Keywords:** Glucose intolerance, Diet, Myocardial perfusion and function, Contrast echocardiography, Hyperaemia

## Abstract

**Background:**

Glucose intolerance is a major health problem and is associated with increased risk of progression to type 2 diabetes mellitus and cardiovascular disease. However, whether glucose intolerance is related to impaired myocardial perfusion is not known. The purpose of the present study was to study the effect of diet-induced glucose intolerance on myocardial function and perfusion during baseline and pharmacological induced hyperaemia.

**Methods:**

Male Wistar rats were randomly exposed to a high fat diet (HFD) or control diet (CD) (n = 8 per group). After 4 weeks, rats underwent an oral glucose tolerance test. Subsequently, rats underwent (contrast) echocardiography to determine myocardial function and perfusion during baseline and dipyridamole-induced hyperaemia (20 mg/kg for 10 min).

**Results:**

Four weeks of HFD feeding resulted in glucose intolerance compared to CD-feeding. Contractile function as represented by fractional shortening was not altered in HFD-fed rats compared to CD-fed rats under baseline conditions. However, dipyridamole increased fractional shortening in CD-fed rats, but not in HFD-fed rats. Basal myocardial perfusion, as measured by estimate of perfusion, was similar in CD- and HFD-fed rats, whereas dipyridamole increased estimate of perfusion in CD-fed rats, but not in HFD-fed rats. However, flow reserve was not different between CD- and HFD-fed rats.

**Conclusions:**

Diet-induced glucose intolerance is associated with impaired myocardial function during conditions of hyperaemia, but myocardial perfusion is maintained. These findings may result in new insights into the effect of glucose intolerance on myocardial function and perfusion during hyperaemia.

## Background

Glucose intolerance defines the intermittent stage between transition from normal glucose levels to type 2 diabetes mellitus [1]. Glucose intolerance is a predictor of cardiovascular disease [2,3] and known to associate with vascular dysfunction and consequent impairment of organ perfusion as one of the appearing consequences [4]. Myocardial perfusion in combination with myocardial performance plays a central role in the balance between myocardial energy supply and demand. Under physiological conditions, myocardial blood flow and function are in balance [5], while pathophysiological conditions leading to vascular dysfunction, such as glucose intolerance, could alter balance between energy supply and demand. Although glucose intolerance-induced vascular dysfunction may impair organ perfusion, data elucidating the effects of glucose intolerance on the perfusion of vital organs like the heart are limited. Up to now, only one group showed that insulin resistance in prediabetic patients was associated with myocardial perfusion defects [6,7].

Contrast echocardiography is a non-invasive method for assessment and quantification of myocardial perfusion that is clinically applied for specific indications in the cardiology setting. Using this technique it has been shown that myocardial blood flow reserve, as a measure for vascular function, is reduced in type 1 diabetic rats [8] and type 1 diabetic patients [9] when compared to normoglycaemic controls. There are currently limited data available on myocardial perfusion in glucose intolerance. In the present study we therefore studied myocardial function and perfusion under baseline and pharmacological-induced hyperaemic conditions in a rat model for diet-induced glucose intolerance using contrast echocardiography. Our hypothesis is that glucose intolerance reduces the myocardial vasodilating capacity, which blunts the increase in myocardial perfusion during hyperaemia. This study demonstrates that diet-induced glucose intolerance is associated with impaired myocardial function during conditions of hyperaemia, but not myocardial perfusion.

## Methods

### Animals and experimental set-up

All experiments were approved by the Institutional Animal Care and Use Committee of the VU University, and were conducted following the European Convention for the Protection of Vertebrate Animals used for Experimental and Other Scientific Purposes [10]. The performed research is in compliance with the modern ARRIVE guidelines on animal research [11].

Adult male Wistar rats (n = 16; body weight 262 ± 1 g; Harlan CBP, Horst, the Netherlands) were fed a high fat diet (HFD) for a period of 4 weeks (n = 8). Animals that received a diet low in fat and sugars (control diet; CD) for 4 weeks served as controls (n = 8). Animals were housed in a temperature-controlled room (20-23 °C; 40-60% humidity) under a 12/12 h light/dark cycle starting at 6.00 am. After 4 weeks, rats underwent an oral glucose tolerance test and (contrast) echocardiography during baseline and after dipyridamole infusion.

### Diets

High fat diet was obtained from Research Diets (D12451, New Brunswick, NJ). The HFD consisted of 24 wt% protein, 24 wt% fat and 41 wt% carbohydrates (8.5 wt% starch, 20.1 wt% sucrose). Control diet (Teklad 2016) was obtained from Harlan (Horst, The Netherlands) and consisted of 17 wt% protein, 4 wt% fat and 61 wt% carbohydrates (45.1 wt% starch, 5.0 wt% sucrose).

### Oral glucose tolerance test

Rats fasted overnight received an oral glucose load (2 g/kg of body weight). Blood glucose levels were measured from tail bleeds with a Precision Xceed Blood Glucose monitoring system (MediSense, UK) before (0) and 15, 30, 60, 90 and 120 min after glucose ingestion [12]. At similar time points, plasma insulin (LINCO research, St. Charles, Missouri) levels were measured as described previously [12].

### Plasma measurements

Plasma haematocrit levels were determined using microcentrifugation. Plasma free fatty acids (WAKO NEFA-C, Wako Pure Chemical Industries, Osaka, Japan), plasma high-density lipoprotein (HDL) cholesterol and plasma low-density lipoprotein (LDL)/very-low-density lipoprotein (VLDL) cholesterol (Abcam, Cambridge, MA) were measured after overnight fasting as previously described [13,14].

### Cannulation of the jugular vein

**Figure 1:**
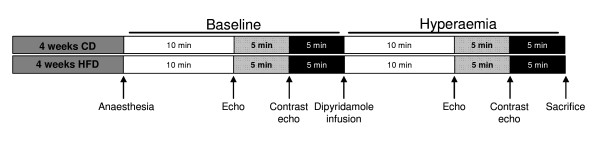
**Protocol (contrast) echocardiography.** After 4 weeks of control diet (CD) or high fat diet (HFD) feeding, rats received anaesthesia for cannulation of the jugular vein followed by echocardiography (echo) and contract echocardiography (contrast echo) during baseline and dipyridamole-induced hyperaemia.

For infusion of the contrast agent for echocardiography, a catheter was placed in the jugular vein under S-Ketamine (Ketanest®, 150 mg/kg, Pfizer, the Netherlands) and Diazepam (3 mg/kg, Centrafarm, the Netherlands) anaesthesia intraperitoneally. After surgery, echocardiography and contrast echocardiography to determine myocardial function and perfusion, respectively, were performed during baseline and dipyridamole-induced hyperaemia (20 mg/kg for 10 minutes) (Figure1).

### Echocardiography

Echocardiography (Siemens, ACUSON, Sequoia 512) was performed as previously described [14]. Briefly, wall thickness (WT) and LV-dimensions (D) during end-systole (ES) and end-diastole (ED) were determined in the M- (motion) mode of the parasternal short-axis view at the level of the papillary muscles. Left ventricular contractile function was calculated by the fractional shortening = (EDD-ESD)/EDD·100%. Analyses were performed off-line (Image-Arena 2.9.1, TomTec Imaging Systems, Unterschleissheim/Munich, Germany). All parameters were averaged over at least three cardiac contractile cycles.

### Myocardial contrast echocardiography

Contrast echocardiography was performed using a Siemens (ACUSON, Sequoia 512) equipped with a 14 MHz linear array transducer (Philips Healthcare, Best, The Netherlands). The contrast agent Sonovue® (Bracco Imaging, Italy), which contains 2x10^8^-5x10^8^/ml sulphur hexafluoride-filled, phospholipid-coated microbubbles with a diameter of 1–10 μm, was prepared according the manufacturer’s instructions and diluted twice by adding 5 ml NaCl (0.9%). Microbubbles were continuously infused into the jugular vein with a rate of 600 μl/min using a dedicated syringe pump (Vueject, Bracco SA, Switzerland). After two minutes of microbubble infusion, perfusion images were taken of the short axis view of the left ventricle at the level of the papillary muscles.

Low acoustic power (mechanical index [MI] 0.20 max) was used for microbubble detection. A perfusion sequence consisted of about 10 cardiac cycles of low MI imaging, followed by a burst of high acoustic power (MI 1.8) for complete contrast destruction. Subsequently, on average 20 cardiac cycles of low MI images were acquired at a frame rate of 14 Hz to allow contrast replenishment in the myocardium. All data were stored for offline analysis.

### Myocardial contrast echocardiography analysis

**Figure 2:**
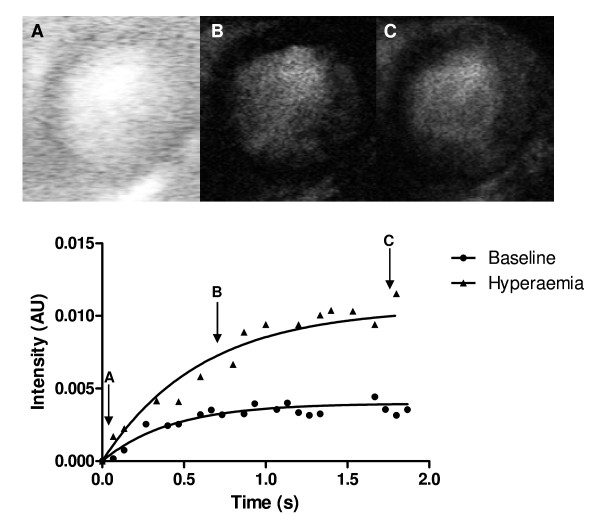
Typical example of contrast echocardiography with replenishment curve in rats.

Custom-designed software was used for analysis of the estimate of perfusion (Matlab, 7.10, R2010A, MathWorks Inc. Massachusetts, USA) with special thanks to R. Meijer. For each cardiac cycle the end-systolic frame was selected manually and regions of interest were drawn in the posterior wall in the short axis view of the left ventricle. Myocardial signal intensity extracted from the frames before microbubble destruction were used to calculate myocardial plateau intensity *A*. Myocardial signal intensities from the frames after microbubble destruction were corrected for background noise by subtracting the signal intensity of the first frame after microbubble destruction (Y_0_). These intensities were then fitted (Y = Y_0_ + (*A*-Y_0_)*(1-exp^(−*ß* *x)^)) for calculation of *ß*, which was transformed into min^-1^ for further analysis (Figure2). The estimate of perfusion was calculated as *A* * *ß.* The flow reserve was calculated as the ratio of hyperaemic and baseline estimate of perfusion.

### Statistical analysis

All data are presented as mean ± SEM. Between group comparisons (CD vs. HFD) were performed using a student *t*-test, whereas baseline vs. dipyridamole intervention was tested with a paired student *t*-test. Oral glucose tolerance test was tested with one-way ANOVA with repeated measurements and Bonferroni post-hoc test. p < 0.05 was considered as statistically significant.

## Results

### Short-term high fat diet feeding results in mild glucose intolerance

**Figure 3:**
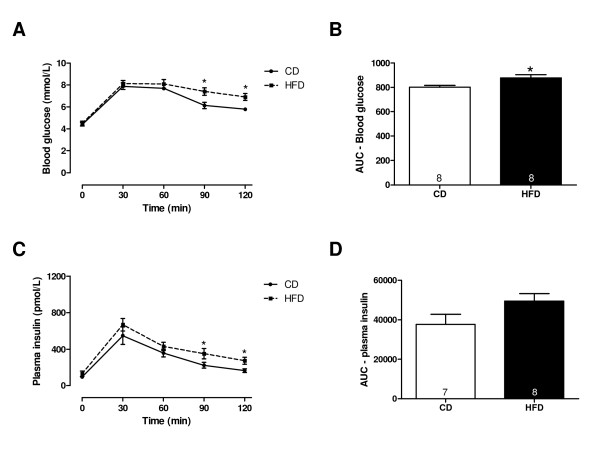
**HFD induced mild glucose intolerance.** Blood glucose (**A,B**) and plasma insulin levels (**C,D**) following an oral glucose load in rats fed a control diet (CD) or high fat diet (HFD). Data are expressed as mean ± SEM, n = 7-8. One-way ANOVA with repeated measurements and Bonferroni post-hoc test (**A,C**) or student *t*-test (**B,D**), * p < 0.05 vs. CD

Characteristics of rats after the diet intervention are summarised in Table1. HFD feeding did not affect body weight, but increased perirenal and epidydimal fat weight. No changes were found in heart weight and tibia length between groups. Haematocrit, blood glucose, plasma insulin, free fatty acid and LDL/VLDL cholesterol levels were unaltered, whereas plasma HDL cholesterol levels were significantly decreased compared to controls. Post-load blood glucose levels were increased in HFD-fed rats compared to CD-fed rats, whereas post-load plasma insulin levels remained unchanged after HFD and CD feeding (Figure3), indicating mild glucose intolerance. 

**Table 1:** Characteristics after short-term diet intervention

	**CD**	**HFD**
**Body composition**		
Body weight (g)	400 ± 6	408 ± 3
Perirenal fat (g)	6.3 ± 0.5	7.5 ± 0.4
Epidydimal fat (g)	7.4 ± 0.4	9.4 ± 0.5 *
Heart weight (g)	1.22 ± 0.05	1.31 ± 0.06
Tibia length (cm)	3.97 ± 0.05	3.89 ± 0.05
**Blood/plasma characteristics**		
Hematocrit (%)	45.2 ± 0.7	44.8 ± 0.7
Blood glucose (mmol/L)	4.4 ± 0.2	4.5 ± 0.2
Plasma insulin (pmol/L)	95.2 ± 14.1	130.8 ± 29.1
Plasma free fatty acid levels (mmol/L)	1.22 ± 0.07	1.09 ± 0.07
Plasma HDL cholesterol (mg/dL)	318.7 ± 13.8	242.6 ± 15.4 *
Plasma LDL/VLDL cholesterol (mg/dL)	7.73 ± 1.23	.45 ± 0.53

Data are mean ± SEM, n = 8, student *t*-test, * p < 0.05 vs. CD. CD, control diet; HFD, high fat diet.

### High fat diet feeding impaired contractile function during dipyridamole-induced hyperaemia

Table2 shows a summary of the echocardiographic parameters measured after 4 weeks of HFD feeding during baseline and dipyridamole-induced hyperaemia. HFD feeding resulted in decreased lumen diameter during end diastole at baseline and reduced systolic wall thickness after dipyridamole infusion. Dipyridamole infusion increased systolic and diastolic wall thickness in CD-fed rats, whereas in HFD-fed rats the end diastolic lumen diameter was increased.

**Table 2:** Echocardiographic parameters after short-term diet intervention

	**CD**		**HFD**	
	**Baseline**	**Hyperaemia**	**Baseline**	**Hyperaemia**
ED lumen diameter (mm)	7.40 ± 0.20	7.46 ± 0.14	6.50 ± 0.25 *	7.07 ± 0.30 ^#^
ES lumen diameter (mm)	3.18 ± 0.21	2.58 ± 0.20	2.99 ± 0.28	2.89 ± 0.19
Diastolic WT (mm)	1.30 ± 0.05	1.41 ± 0.06 ^#^	1.34 ± 0.09	1.42 ± 0.04
Systolic WT (mm)	2.96 ± 0.15	3.40 ± 0.06 ^#^	2.85 ± 0.134	3.10 ± 0.10 *

Data are mean ± SEM, n = 8, student *t*-test, * p < 0.05 diet effect, ^#^ p < 0.05 hyperaemia effect. *CD*, control diet; *HFD*, high fat diet; *ED*, end-diastolic; *ES*, end-systolic; *WT*, wall thickness.

**Figure 4:**
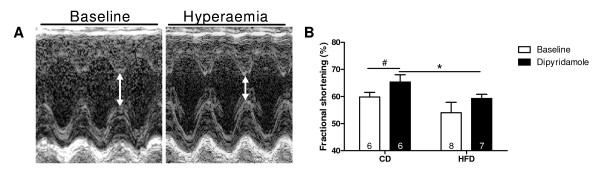
**Contractile function during baseline and dipyridamole-induced hyperaemia.** Representative examples of the m-mode of the left ventricle at the level of the papillary muscle (**A**). Contractile function, as represented by the fractional shortening, measured in rats fed a control diet (CD) and high fat diet (HFD) for 4 weeks during baseline and dipyridamole-induced hyperaemia (**B**). Data are expressed as mean ± SEM, n = 6-8. (paired) student *t*-test, * p < 0.05 diet effect, # p < 0.05 hyperaemia effect.

Contractile function, as represented by fractional shortening, was unaffected by HFD feeding under baseline conditions (Figure4). During dipyridamole infusion, fractional shortening was increased in CD-fed rats, but not in HFD-fed rats, whereas fractional shortening was even decreased in HFD vs. CD-fed rats, suggesting impaired contractile function during dipyridamole-induced hyperaemia.

### Unchanged myocardial perfusion after high fat diet feeding

**Figure 5:**
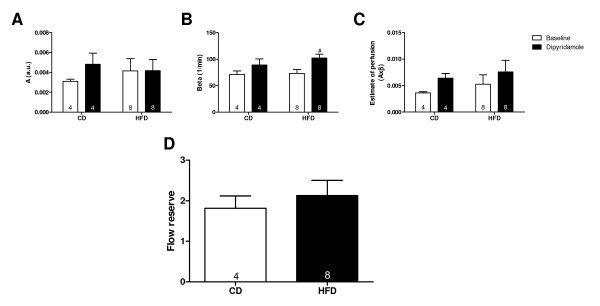
**Myocardial perfusion measured with contrast echocardiography.** Plateau intensity *A* (**A**), beta β (**B**), estimate of perfusion (**C**) and flow reserve (**D**) in rats fed a control (CD) or high fat diet (HFD) measured during baseline and dipyridamole-induced hyperaemia. Data are expressed as mean ± SEM, n = 4-8. (paired) student *t*-test, ^#^ p < 0.05.

HFD-feeding did not alter plateau intensity *A*, β and estimate of perfusion compared to CD-fed rats (Figure5A-C). Dipyridamole infusion increased plateau intensity *A*, β and estimate of perfusion in CD-fed rats (Figure5A-C), however, failed to reach significance. In HFD-fed rats, only β was significantly increased after dipyridamole infusion (Figure5A-C). No differences were found in flow reserve between CD- and HFD-fed rats (Figure5D), suggesting unaltered myocardial perfusion after 4 weeks of HFD feeding.

## Discussion

In the present study, we examined the effect of glucose intolerance on myocardial function and perfusion. We found that short-term high fat diet feeding resulted in glucose intolerance without affecting myocardial function and perfusion under baseline conditions. Diet-induced glucose intolerance impaired myocardial contractile function during conditions of hyperaemia, in the absence of alterations in myocardial perfusion.

The present study showed that short-term high fat diet feeding resulted in glucose intolerance without obesity affecting myocardial contractile function under resting conditions. Our results confirm previous findings, showing that 4 weeks of high fat diet feeding in rats resulted in glucose intolerance without affecting function [15], whereas 8 weeks of high fat diet feeding resulted in glucose intolerance with mildly impaired contractile function [15]. One of the suggested mechanisms that may contribute to alterations in myocardial blood flow during prediabetes or diabetes are changes in nitric oxide availability. High fat diet feeding in male rats decreased nitric oxide availability [16], whereas increased nitric oxide bioavailability in eNOS−/− mice has been shown to attenuate high fat diet-induced metabolic alterations associated with insulin resistance [17]. Taken together, these results suggest that 4 weeks of high fat diet feeding in a rat results in a mild model of glucose intolerance without impaired myocardial function.

Under physiological conditions, myocardial perfusion and function are in balance [5]. However, a perfusion-contraction mismatch might exist in glucose intolerance. In the present study, diet-induced glucose intolerance did not affect myocardial perfusion measured with contrast echocardiography during baseline conditions. These results are in agreement with Menard *et al.*[18], who found no differences in myocardial perfusion index measured by ^13^ N] ammonia PET in rats fed a high fructose and high fat diet with additional streptozotocin. Accordingly, in patients with insulin resistance or glucose tolerance it was found that insulin resistance was associated with a defect in myocardial perfusion measured by single photon emission computed tomography, which was independent of glucose tolerance and obesity [6]. Together, these results suggest that under baseline conditions diet-induced glucose intolerance does not affect myocardial function and perfusion.

Dipyridamole is used to pharmacologically induce maximal vasodilation. Dipyridamole blocks the uptake of adenosine, thereby increasing the circulating levels of adenosine, which results in maximal coronary blood flow due to decreased coronary vascular resistance [19]. During hyperaemia, myocardial contractile function was increased in healthy rats, but not in rats with diet-induced glucose intolerance. On the contrary, this hyperaemic-induced increase in contractile function was not seen in healthy and type 1 diabetic rats [8]. Besides glucose intolerance, several variables could be associated with alterations in myocardial function. Our experimental rat model with high fat diet feeding resulted in development of glucose intolerance, but rats did not become obese. Consequently, obesity can not be explanatory for the findings of the effect of high fat diet feeding on myocardial function.

Myocardial blood flow reserve is the increase in blood flow that can be achieved from basal perfusion to maximal vasodilatation, and is therefore a measurement of the ability of the microvasculature to respond to an increase in oxygen demand. In type 1 diabetic rats [8] and patients [9] it was shown that myocardial blood flow reserve measured with contrast echocardiography was decreased compared to healthy controls. In the present study, myocardial blood flow reserve was unchanged between healthy rats and rats with glucose intolerance, suggesting that glucose intolerance does not reduce the vasodilating capacity of the myocardium. Increases in myocardial contractile function will lead to a metabolically-mediated increase in myocardial blood flow, however, an increase in coronary perfusion does not have to increase myocardial contractile function [5], which might explain the hyperaemic-induced differences in myocardial function and perfusion in rats with glucose intolerance.

A limitation of this study is the small amount of rats used. Although this is a pilot study, ideally, this data set should have been analysed with a two-way ANOVA with Bonferronni post-hoc testing for multiple group comparisons.

## Conclusions

In conclusion, the present data demonstrate that diet-induced glucose intolerance is associated with impaired myocardial function during conditions of hyperaemia, but not myocardial perfusion. These findings may result in new insights into the effect of glucose intolerance on myocardial function and perfusion during hyperaemia.

## Abbreviations

*A*: Myocardial plateau intensity; ß: beta; CD: Control diet; D: LV-dimensions; ED: End-diastole; ES: End-systole; HDL: High-density lipoprotein cholesterol; HFD: High fat diet; LDL: Low-density lipoprotein cholesterol; M: Motion; MI: Mechanical index; VLDL: Very-low-density lipoprotein cholesterol; WT: Wall thickness; Y_0_: Signal intensity of the first frame after microbubble destruction.

## Competing interests

The authors declare that they have no competing interests.

## Authors’ contributions

CEvdB participated in performing the study, data analysis, statistics and writing the manuscript. CSEB in part performed the study and supported the data analysis. MBK in part performed the study. SAL reviewed/edited the manuscript. CB participated in the design of the study and reviewed/edited the manuscript. RAB supervised the study, participated in the design of the study and reviewed/edited the manuscript. All authors read and approved the final manuscript.
